# Refusal of Food and Forcible Feeding

**Published:** 1908-06-06

**Authors:** 


### Mental Diseases.

### REFUSAL OF FOOD AND FORCIBLE FEEDING.

Persistent refusal of food may become a symptom demanding special treatment in both early and developed cases of mental disease. It may occur in every class of case, but is most frequently seen in states of mental depression. The reason for the refusal may be merely lack of appetite and disinclination for food, due to a disordered alimentary system. In severe cases of this kind the offensiveness of the breath is very great, there is much sordes round the teeth and lips, and the patient complains of a disgusting taste in the mouth. Food or the thought of it makes him heave, and the reason for refusal is plain. A simple enema at once, five grains of calomel or a blue pill at night followed by a saline purge in the morning, the pill and saline being repeated daily until the bowel has been cleared of any fæcal accumulation, will usually suffice for treatment. Should the offensiveness of breath and foul taste in the mouth be great, a solution of chlorine and oxygen (that sold as Hermitine is excellent) should be used every hour or two as a mouthwash, and the patient told to swallow some every time it is used. In other cases refusal of food is due to delusions of various kinds, and forcible feeding may become necessary. Should these cases be left they will often either starve to death or to such a weakened condition that there will be grave danger to life. Cases of acute mania refuse food on account of their restless and talkative condition; they appear too busy to spare time to take it; or they have some temporary delusion or hallucination which is the cause. These cases, if their bodily condition does not forbid, may be left for a short time, and will usually be induced to eat. In puerperal cases the rule is to feed them early, especially the melancholics.

Forcible feeding may be performed by the nasal or by the oral route. In either case a soft red rubber tube should be used, which should always be stretched and examined before using to make sure that it is sound and has not perished. Whichever method be adopted, let the tube be as large as possible. For nasal feeding Jaques' red rubber œsophageal tube, No. 16, will, as a rule, pass easily, and No. 18 can often be passed without difficulty. For oral use No. 28, or the extra large size, No. 30, should be used, and will only in very rare cases be found too large. The reasons for using large tubes are that they usually pass more readily; they cannot be passed through the glottis, as may happen with a small nasal tube; they fill the œsophagus better and prevent regurgitation by the side of the tube; they have more substance, and therefore do not kink as easily as the smaller ones; and they have a bigger calibre, which allows the feeding to be more quickly accomplished. A large vulcanite funnel is inserted into the end of the tube. The most useful gag is a wedge screw gag, with some indiarubber tubing over the blades to prevent injury to the teeth. In oral feeding the tube should be dipped in the food before passing, in order to lubricate it; but for the nasal tube oil or vaseline must be used and the entire tube lubricated, since ulceration of the nose is easily produced if the tube is big and not well lubricated. Nasal feeding, as a rule, involves less struggling, no need for gagging, less danger of the patient biting the tube and swallowing a piece, and less regurgitation than the oral method; but, on the other hand, the food does not so readily flow down the smaller calibre of the tube, and the moral effect is not so great. Any warm fluid food may be used, and a little salt should always be added; sometimes excess is put in to induce thirst and so cause the patient to take naturally, but this is not always to be recommended. It is a good plan when feeding by the oral method to wash out the stomach with a solution of Hermitine before each feed, the stomach being thus kept sweet, and foulness of breath abolished.

The patient may be fed either sitting up or lying down, the latter being the usual position; but whichever is adopted plenty of assistants are necessary to hold the patient. For the sitting position a high-backed chair is required, and towels are used as means of restraint. To feed lying down the patient is placed on a bed, a small hard bolster being put under the head. No tight clothing must constrict the throat, chest, or abdomen. A jack-towel, folded lengthwise, is placed round the legs, directly over or just above the knees. A nurse kneels on each end of the towel and holds the patient's hand of the corresponding side. Another nurse takes a hand towel folded lengthwise, places it over the patient's forehead and holds it on each side firmly down to the bolster. An assistant must never be allowed to kneel on the patient, press on the chest or abdomen, or hold the patient's head by the hair. The gag is inserted, opened wide, and firmly held by another assistant. The tube is then passed and the feeding accomplished. Whilst withdrawing the tube it should be compressed to prevent drops leaving the end as it passes over the epiglottis and entering the larynx. In nasal feeding care must be taken that the tube does not pass into the mouth and coil up there.

Of the accidents that happen during forcible feeding the worst are food passing the larynx and a piece of tube being bitten off and swallowed. In old-standing and in severe cases the laryngeal reflex is often much diminished or absent. To prevent the former accident use a large tube, make sure it is in the stomach before pouring in food, let it empty before withdrawing, and compress it whilst so doing. To prevent the latter, make sure before starting that the tube is good and sound, and see that the head and gag are firmly held. If in feeding it is found that there is not help enough at hand, wait until more can be obtained. Voluntary vomiting after each feed is usually stopped by threatening to keep what is vomited and to feed again at once with it. If the threat is not sufficient, carrying it once into effect seldom fails.

